# Strongylids of Domestic Horses in Eastern Slovakia: Species Diversity and Evaluation of Particular Factors Affecting Strongylid Communities

**DOI:** 10.1007/s11686-024-00854-7

**Published:** 2024-05-22

**Authors:** Tetiana A. Kuzmina, Alzbeta Königová, Ludmila Burcáková, Michal Babjak, Yaroslav Syrota

**Affiliations:** 1 Institute of Parasitology, Slovak Academy of Sciences, Hlinkova 3, 04001 Kosice, Slovakia; 2 I. I. Schmalhausen Institute of Zoology NAS of Ukraine, Bogdan Khmelnytsky street, 15, Kyiv, Ukraine; 3 University of Veterinary Medicine and Pharmacy in Kosice, Komenskeho 73, 04181 Kosice, Slovakia

**Keywords:** Nematoda, Strongylidae, Parasite community, Horse-management conditions, FECRT, Slovakia

## Abstract

**Purpose:**

This study aimed to examine the species diversity and structure of the strongylid community in domestic horses in Eastern Slovakia. Also, an analysis of the impact of age, sex, and collection location factors on the strongyid communities was performed.

**Methods:**

Twenty-seven horses 1.5–21 years old from two farms in eastern Slovakia with different horse-management conditions were studied. Strongylids were collected after horse treatments with Noromectin (0.2 mg ivermectin); 66,170 specimens were collected and identified. Faecal egg count reduction test (FECRT) was performed following fenbendazole (FBZ) and ivermectin (IVM) treatments.

**Results:**

Twenty-four strongylid species were found; horses were infected with 6 to 16 (average = 11.7) species. Six cyathostomin species (*Cylicocyclus nassatus*, *Cyathostomum catinatum*, *C. pateratum*, *Cylicostephanus longibursatus*, *C. goldi*, *C. calicatus*) were the most prevalent; *C. catinatum* was the dominant species in both farms (Berger-Parker index 0.34 and 0.42). The structure of the strongylid community was multimodal with dominant, subdominant, background, and rare species. The Mantel test showed that horse age and sex did not significantly affect the nematode infracommunity composition (*p* > 0.05), while the impact of the collection location (farm) was significant (*p* = 0.03). Additionally, *C. longibursatus* was identified as the species contributing significantly to the observed farm differences. Strong resistance to FBZ was documented on both farms (FECRT was 36.4% and 22.7%); IVM resistance was not observed (FECRT = 100%).

**Conclusion:**

This study presents the first report on the strongylids parasitizing domestic horses in Eastern Slovakia and gives basic information for further studies of horse parasites and their control in the region.

**Supplementary Information:**

The online version contains supplementary material available at 10.1007/s11686-024-00854-7.

## Introduction

Among more than 90 species that parasitize equids [1], strongylid nematodes are by far one of the most widespread and pathogenic group of parasites [2–6]. Strongylids parasitize all wild and domestic equids worldwide and can cause severe health problems, including loss of weight, colic, watery diarrhoea and ventral oedema, decreasing the productivity and breeding qualities of horses [7–10]. The pathogenic influence of different strongylids species in the horse is different; large strongylids (subfamily Strongylinae), especially representatives of the genus *Strongylus*, are the most pathogenic because they have complex tissue migrations through different abdominal organs over several months causing severe tissue damage and can lead to life-threatening complications [11–14]. Pathogenicity of small strongylids (subfamily Cyathostominae) is not as severe; usually, cyathostomin infection in horses is asymptomatic. However, synchronous emerging of cyathostomin parasitic larvae from the intestinal wall can cause a life-threatening syndrome known as ‘‘larval cyathostominosis’’ with a case-fatality rate reported up to 50% [10, 15, 16].

An individual horse may be co-infected with as many as 15–25 species of strongylids [5, 6, 17–19]. Accordingly, the horse strongylid community has a complex structure [5, 8; 20–25]. The prevalence and proportion of each species in the strongylid community may vary depending on the age, sex, and horse-management conditions, in particular, the frequency of anthelmintic treatments [5, 22]. The proportion of large strongylids in the communities of domestic horses is remarkably low– from less than 1–2% [5, 23]; small strongylids (Cyathostominae) comprise more than 95% of the overall strongylid communities. In recent decades, due to the frequent use of highly effective anthelmintics, the proportion of large strongylids in the communities has been decreasing, and cyathostomins have become the main group of horse parasites [2, 4, 6, 26–30]. Moreover, cyathostomins have developed resistance to various groups of anthelmintics worldwide [27; 31–33]. It has been shown that not all strongylid species were found to be resistant; so far, resistance to benzimidazole (BZ) drugs has been documented for 10–11 cyathostomin species [34–38], while seven cyathostomin species demonstrated ivermectin (IVM) resistance [35]. Therefore, for effective parasitological monitoring and the elaboration of efficient parasite control methods, it is imperative to know the species composition of the strongylid community on particular horse farms.

Of 64 strongylid species described to date [3], domestic horses are parasitized by more than 40 species [3, 5, 8]. Meta-analysis of published data collected worldwide revealed that three cyathostomin species (*Cylicocyclus nassatus*, *Cylicostephanus longibursatus*, and *Cyathostomum catinatum*) were the most prevalent and relatively abundant species of equine strongylids [6]. Simultaneously, the species composition of strongylid communities varies in different countries [25]. In Europe, horse strongylid studies have been performed in different countries for more than 50 years [5, 8, 20, 22, 23, 28–30, 40–44]. Despite the horse industry being widely developed in the Slovak Republic, where more than 22,500 horses of 40 breeds are kept and bred [45], studies of the species diversity and structure of strongylid communities have not been carried out. In the late 1950s, a study of strongylids was performed in former Czechoslovakia [40]; as a result, 35 strongylid species were found in domestic horses. Later, a study of equine strongylids in Slovakia was conducted using the Reverse line blot hybridization assay for the species-specific identification of cyathostomins [46]. Through this method, 13 cyathostomin species were detected in faecal samples from domestic horses; information on the prevalence or proportion of these species in the strongylid community has not been documented. Several studies of strongylids in Slovak horse farms have been carried out using coprological methods, presumably to examine the distribution of anthelmintic resistance [47–51]. However, an analysis of all available literature sources revealed that data on the species composition of the equine strongylid communities in Slovakia have not been published to date. This study aimed to examine the species diversity and structure of the strongylid community in domestic horses in Eastern Slovakia, along with evaluating the impact of various factors, including age, sex, and the collection location (farm with different horse-management conditions), on the strongylid communities. Furthermore, investigating the efficacy of anthelmintic treatment and the presence of anthelmintic resistance emerged as an additional area of interest within the scope of this research.

## Materials and methods

### Locations and Horses Examined

The study was conducted in April–May and August 2023 in two horse farms in Eastern Slovakia, each having different horse-management conditions. Farm #1 had 20 horses used for sport and recreational riding. The horses were housed in stables and had limited access to small individual paddocks (0.2–0.4 ha) for 4–6 h per day. At this farm, regular stable cleaning and regular deworming of all horses 2–3 times per year were practised. Farm #2 had about 40 horses used for breeding; these horses were kept in stables for 8–12 h at night and had access to grazing on large permanent pastures (20.0–29.9 ha) for 12–16 h per day. At this farm, irregular stable cleaning and irregular deworming of all horses two times per year were practised.

Twenty-seven horses 1.5–21 years old were used for the study: nine horses from Farm #1 and 18 from Farm #2 (Table 1). All horses had not been dewormed with any anthelmintic drugs for more than three months before the experimental treatment. Clinical signs of parasitoses were not observed in the horses before and during the study.

**Table 1:** Details on the horses from two Slovakian farms included in the study

Variable	Categories	Farm #1 (*n* = 9)	Farm #2 (*n* = 18)
Age	min–max (average ± SD)	3–21 (8.2 ± 6.1)	1.5–14 (4.4 ± 3.4)
	Young (1–<4 years)	3 (33.33%)	13 (72.22%)
	Adult (4–15 years)	4 (44.44%)	5 (27.78%)
	Old (> 15 years)	2 (22.22%)	0
Gender	Females	5 (55.6%)	9 (50.0%)
	Males	4 (44.44%)	9 (50.0%)
Breed	Warmblood horses	9 (100.0%)	—
	Shagya-Arabian	—	18 (100.0%)
Horse use	Sport	9	—
	Breeding	—	18
Pasture/paddock	Size	individual paddocks (0.2–0.4 ha)	permanent pastures (20.0–29.9 ha)
	Cleaning	Yes (once a week)	No
	Access to pasture	4–6 h/day	8–16 h/day
Deworming:	Frequency	2–3 times a year	2 times a year
	Anthelmintic rotation	Yes	Yes
Anthelmintic resistance	Benzimidazoles (fenbendazole)	Yes FECRT* = 36.4%	Yes FECRT = 22.7%
	Ivermectin	No	No

*– FECRT; faecal egg count reduction test (Coles et al., [53])

### Experimental Design: Collection of Strongylid Nematodes

Strongylid nematodes were collected using the in vivo method of diagnostic deworming [44] with a modification. Faecal egg counts (FEC) using the McMaster technique [52] with a sensitivity of 25 eggs per gram of faeces (EPG) were performed for every horse before treatment. Only horses with EPG > 200 were selected for the study.

All 27 horses selected were treated with the macrocyclic lactone drug Noromectin (140 mg/g IVM, Norbrook Laboratories Ltd, Monaghan, Ireland). Faecal samples (500 g per sample) were collected from each horse 24, 36, and 48 h after treatment; because less than 5% of the total nematode number nematodes were expelled from the horse intestine 48 h after treatment, faecal samples were not collected at 60 h and 72 h after treatment. Larger faecal samples– 500 g instead of 200 g in the original description of the method [44], were collected because the samples from horses with low EPG values contained an insufficient number of nematodes. The faecal samples were washed with 0.9% saline and examined under a magnifying glass to collect strongylids. All strongylids expelled were collected manually and fixed in 70% ethanol. Faecal egg counts were performed 14 days after treatment; strongylid eggs were not found in any samples. Thus, all intestinal-lumen stages of strongylids were expelled from the horse intestines and available for examination. All strongylids collected were stored in 70% ethanol, clarified with lactophenol (25% lactic acid, 25% phenol, 25% glycerine, 25% distilled water), and identified under a light microscope using the following morphological criteria: the size of nematode, size and shape of the buccal capsule, mouth collar, cephalic papillae, internal and external leaf-crowns, the shape of extra-chitinous supports external leaf-crown and oesophagal funnel as well as the shape of posterior ends of females and bursa of males [3].

### Faecal Egg Count Reduction Test (FECRT)

The faecal egg count reduction test (FECRT) [53] was used to define the efficacy of anthelmintic treatment and determine the presence or absence of resistance to benzimidazole (BZ) or macrocyclic lactone (ivermectin, IVM) anthelmintics in strongylids. For the FECRT, the faecal egg counts were carried out a day before anthelmintic treatment (Day 0) and ten days (Day 10) for BZ (fenbendazole)-treated horses, or 14 days (Day 14) for IVM-treated horses, after treatment using the McMaster technique [52]. The calculation of FECRT results was performed using the formula of Coles et al. [53]:

$${\rm{FECRT}}\;{\rm{(\% )}}\;{\rm{ = }}\;{\rm{100}}\; \times \;{\rm{(1}}\;{\rm{ - }}\;{\rm{EPGi}}\;{\rm{/}}\;{\rm{EPGt)}}$$

where EPGi was faecal egg count data on Day 0 (before treatment), EPGt– faecal egg count data on Day 10 or 14 (after treatment).

All 27 experimental horses were treated with the BZ drug Panacur (187.5 mg/g fenbendazole, Intervet). This part of the study was performed four months before the strongylid collection. All horses underwent an assessment of IVM efficacy using FECRT simultaneously with the collection of adult strongylids.

### Data Analysis

The prevalence (P) of each strongylid species was separately calculated following the methodology described by Bush et al. [54]. Since the study did not involve horse dissection, we could not use the terms “intensity of infection” or “mean intensity of infection” for our data. The alternative term “mean intensity per sample” was chosen to quantify the number of nematodes in faecal samples from a single horse. Additionally, the proportion of each species in the strongylid community was estimated as the percentage of specimens of particular species in the entire helminth sample. Previous research [5] has revealed substantial differences in the strongylid communities of old horses, specifically those over 15 years, compared with younger horses. Due to the minimal number of old horses in the dataset used for this study (2 horses), they were omitted from this analysis to avoid potential bias. Nonetheless, the relevant sections of the Results detailed observations regarding helminths in the excluded horses.

All data analysis was conducted with the R programming language [55]. The *tidyverse* packages collection was employed for data manipulation and visualization [56]. Initially, univariate statistical tests were conducted to compare helminth community metrics across the two farms. The Wilcoxon rank-sum test was utilized to compare species richness, bootstrap t-tests [57] for the number of strongylid individuals per sample and the EPG, and Hutcheson’s t-test for the Shannon index [58]. The choice of these tests was guided by the non-parametric nature of the data under consideration. After the execution of the individual tests, the resulting p-values were adjusted using the Holm method. Then, the relationship between EPG and the number of strongylid individuals per sample from an individual horse was assessed using Spearman’s rank correlation.

Subsequently, the Mantel tests [59] were used to assess the association between helminth infracommunities and several variables, including the collection location (farm), age, and sex of the horses. Based on the abundance matrix that contained data on the quantity of each helminth species in each horse, a dissimilarity matrix was produced using Bray-Curtis dissimilarity. Afterwards, three Mantel tests were conducted to sequentially compare this matrix with the distance matrices for a collection location (farm), horse age, and sex. The distance matrices for the collection location and sex were computed using Gower’s distance, while Euclidean distance was used for age. Following the Mantel tests, Holm correction was applied to adjust the p-values obtained, ensuring a robust assessment of statistical significance across multiple comparisons. Then, a model-based approach to analyzing the multivariate abundance data [60] was employed to additionally examine the effects of the collection site on the number of different helminth species within horses. An analysis of the variance table was computed using the likelihood ratio test (LRT) to evaluate the statistical significance of the findings. Finally, a non-metric multidimensional scaling (nMDS) analysis was performed to visualize the patterns of helminth community composition across horses [59].

The supplementary material linked to this article includes technical details on the statistical analysis (see Supplementary data).

## Results

### Characteristics of the General Strongylid Sample

All horses were infected with strongylid nematodes; in total, 66,170 specimens were collected in the study. On average, 2,646.8 strongylids were found per sample, with the number of specimens per sample ranging from 24 to 8,245 and a median equal to 2,081. Twenty-four strongylid species from 10 genera were recorded (Table 2), including three species of large strongylids (subfamily Strongylinae) and 21 species of small strongylids (subfamily Cyathostominae). On average, horses were infected with 11.7 species, with the median number of species per horse being 11 and ranging from 6 to 16.

**Table 2:** Prevalence and 95% confidence intervals in square brackets, along with the mean number of strongylid individuals per sample (MN) and their range in round brackets, for 24 strongylid species collected in horses from two farms in Slovakia. (—)– species not found

Helminth species	Farm #1 (*n* = 7)		Farm #2 (*n* = 18)		
	Prevalence, %	MN	Prevalence, %	MN	
**subfamily Strongylinae**					
*Strongylus vulgaris*	—	—	5.56 [0.28–27.14]	2	
*Triodontophorus. serratus*	14.29 [0.73–55.42]	1	[4.7–41.41]	5 (1–12)	
*T. brevicauda*	14.29 [0.73–55.42]	2	—	—	
**subfamily Cyathostominae**					
*Cyathostomum catinatum*	100 [62.29–100]	387.7 (3–2144)	100 [81.47–100]		1363.2 (4–5175)
*C. pateratum*	85.71 [44.58–99.27]	80.5 (4–316)	94.44 [72.86–99.72]		407.7 (9–2793)
*Coronocyclus coronatus*	71.43 [34.13–94.66]	36.2 (2–87)	66.67 [41.41–84.37]		16.8 (2–46)
*С. labiatus*	28.57 [5.34–65.87]	87 (73–101)	61.11 [37.47–81.47]		14.3 (1–38)
*C. labratus*	28.57 [5.34–65.87]	11 (4–18)	38.89 [18.53–62.53]		7.3 (1–26)
*Cylicostephanus calicatus*	100 [62.29–100]	26.4 (1–67)	88.89 [66.97–97.99]		42.6 (1–258)
*C. longibursatus*	100 [62.29–100]	48.9 (2–243)	100 [81.47–100]		350.1 (3–1238)
*C. goldi*	85.71 [44.58–99.27]	14.2 (2–40)	88.89 [66.97–97.99]		95.8 (1–376)
*C. minutus*	42.86 [12.88–77.47]	33.3 (4–60)	94.44 [72.86–99.72]		142 (1–612)
*Cylicocyclus nasssatus*	100 [62.29–100]	365 (4–1461)	100 [81.47–100]		556.6 (6–1772)
*C. insigne*	71.43 [34.13–94.66]	43 (2–116)	55.56 [33.03–76.36]		69.5 (1–304)
*C. leptostomum*	57.14 [22.53–87.12]	204.2 (4–717)	88.89 [66.97–97.99]		142.9 (2–533)
*C. aswhorthi*	42.86 [12.88–77.47]	27 (8–48)	88.89 [66.97–97.99]		141 (2–936)
*C. radiatus*	—	—	5.56 [0.28–27.14]		3
*C. elongatus*	—	—	5.56 [0.28–27.14]		1
*C. brevicapsulatus*	—	—	27.78 [11.64–52.86]		6.6 (1–24)
*Cylicotetrapedon bidentatus*	14.29 [0.73–55.42]	1	—		—
*Petrovinema poculatum*	—	—	44.44 [23.64–66.97]		5 (2–12)
*Poteriostomum imparidentatum*	14.29 [0.73–55.42]	3	5.56 [0.28–27.14]		12
*P. euproctus*	14.29 [0.73–55.42]	1			
*Parapoteriostomum mettami*	57.14 [22.53–87.12]	1.5 (1–2)	38.89 [18.53–62.53]		4.9 (1–12)

**Fig. 1:**
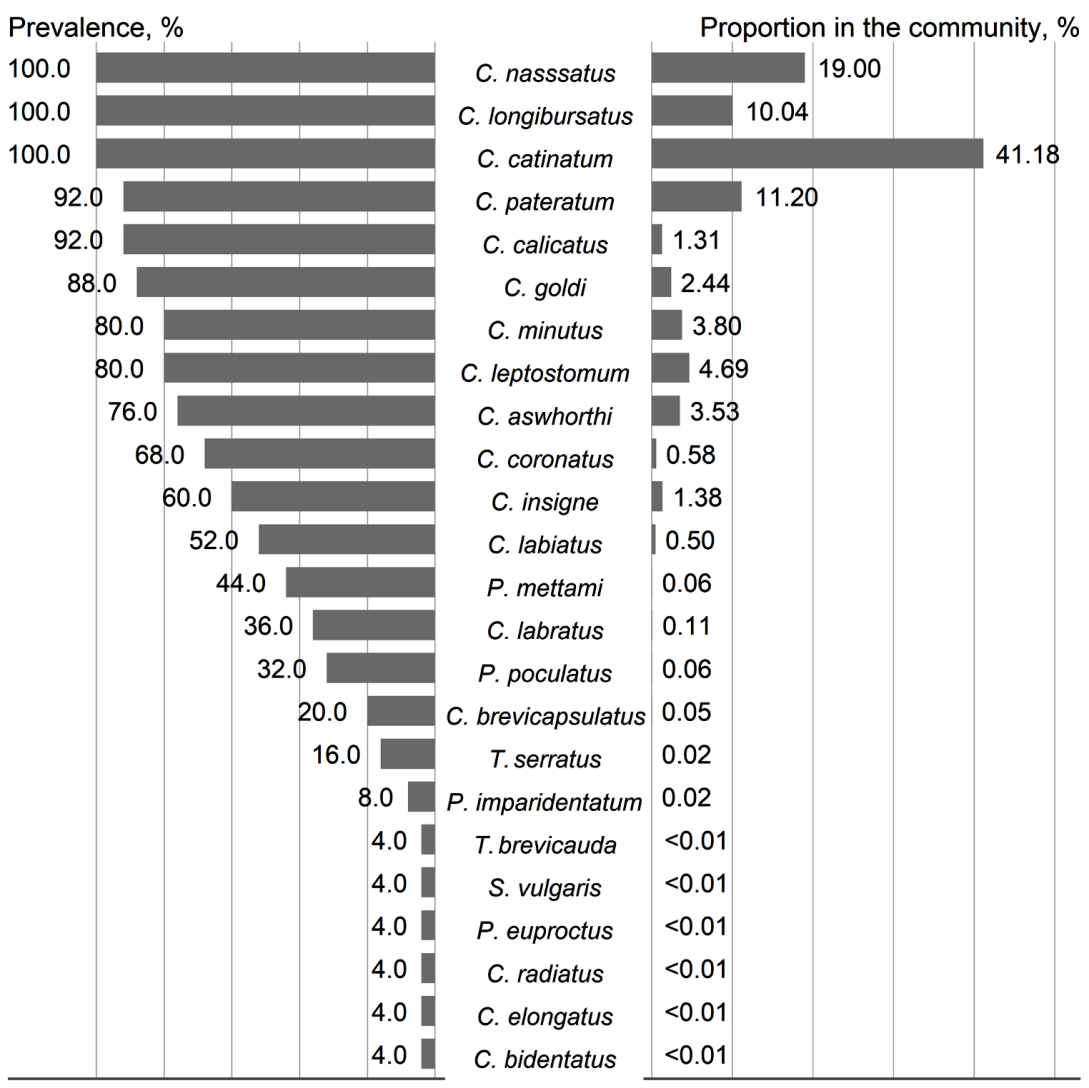
Prevalence (%) and proportion (%) of separate species in the strongylid community of horses from Slovakia

The full dataset, which includes information on two communities, exhibited moderate diversity, as indicated by the Shannon diversity index of 1.83. Six cyathostomin species (*C. nassatus*, *C. catinatum*, *C. longibursatus*, *C. goldi*, *C. calicatus*, and *C*. *pateratum*) were the most prevalent in the sample (Fig. 1). These species accounted for approximately 85% of the total strongylids collected. The most abundant species was *C. catinatum*, with a Berger-Parker dominance index of 0.41.

Additionally, on Farm #1, two horses older than 14 years were found to have very low infection with strongylids; 197 specimens of nine species (*C. nasssatus, C. catinatum, С. leptostomum, C. calicatus, C. aswhorthi, C. longibursatus, C. goldi, C. minutus, C. coronatus*) were collected from these horses.

**Fig. 2:**
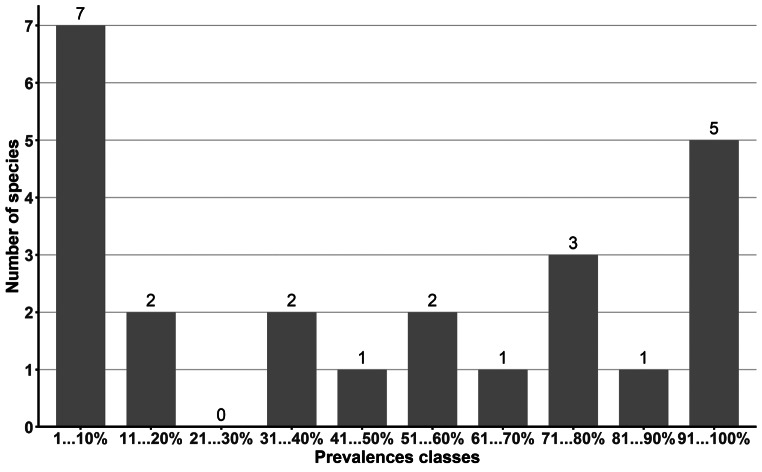
Distribution of 24 strongylid species found in horses from Slovakia into ten prevalence classes

The distribution of 24 strongylid species found in our study in ten prevalence classes revealed that the structure of the strongylid community is multimodal with eight dominant (P > 80–100%), four subdominant (P = > 50–80%), three background (P = > 20–50%) and eight rare ( P≤ 20%) species (Fig. 2).

### Comparison of the Strongylid Communities between Two Farms

On Farm #1, 7,968 strongylid specimens were collected and identified. The community exhibited an average number of strongylid individuals per sample of 1,138.3, with a median of 396, ranging from 24 to 3,569 nematode individuals per sample. Meanwhile, Farm #2 had a more abundant strongylid community. In total, 58,202 specimens were collected. The number of strongylid individuals per sample on this farm varied from 301 to 8,245, with an average of 3,233.4 specimens per sample and a median of 2,295. However, a statistical comparison of the number of strongylid individuals per sample between these two farms showed no significant differences (Bootstrap Welch Two Sample t-test; *p* = 0.074).

Nineteen species of strongylids were documented at Farm #1, whereas Farm #2 showed a marginally higher species count, with 21 strongylids species identified (Table 2). The species richness on Farm #1 averaged 10.4 species per horse, with a median of 8 species and a range from 6 to 16 species. In comparison, Farm #2 demonstrated a higher species richness, averaging 12.2 species per horse, with the median reaching 12.5 species and ranging from 7 to 16 species. The comparison of the species richness between both farms did not show any significant differences (Wilcoxon rank sum test; W = 44.5, *p* = 0.816).

Both farms demonstrated moderate levels of species diversity, with Farm #1 having a Shannon diversity index of 1.80 and Farm #2 slightly higher, at 1.81. Hutcheson’s t-test for comparing the diversity indices yielded no significant difference in species diversity between the farms; t = -0.2, *p* = 0.842. *Cyathostomum catinatum* was identified as the dominant species in both farms, exhibiting a Berger-Parker index of 0.34 in Farm #1 and 0.42 in Farm #2.

**Fig. 3:**
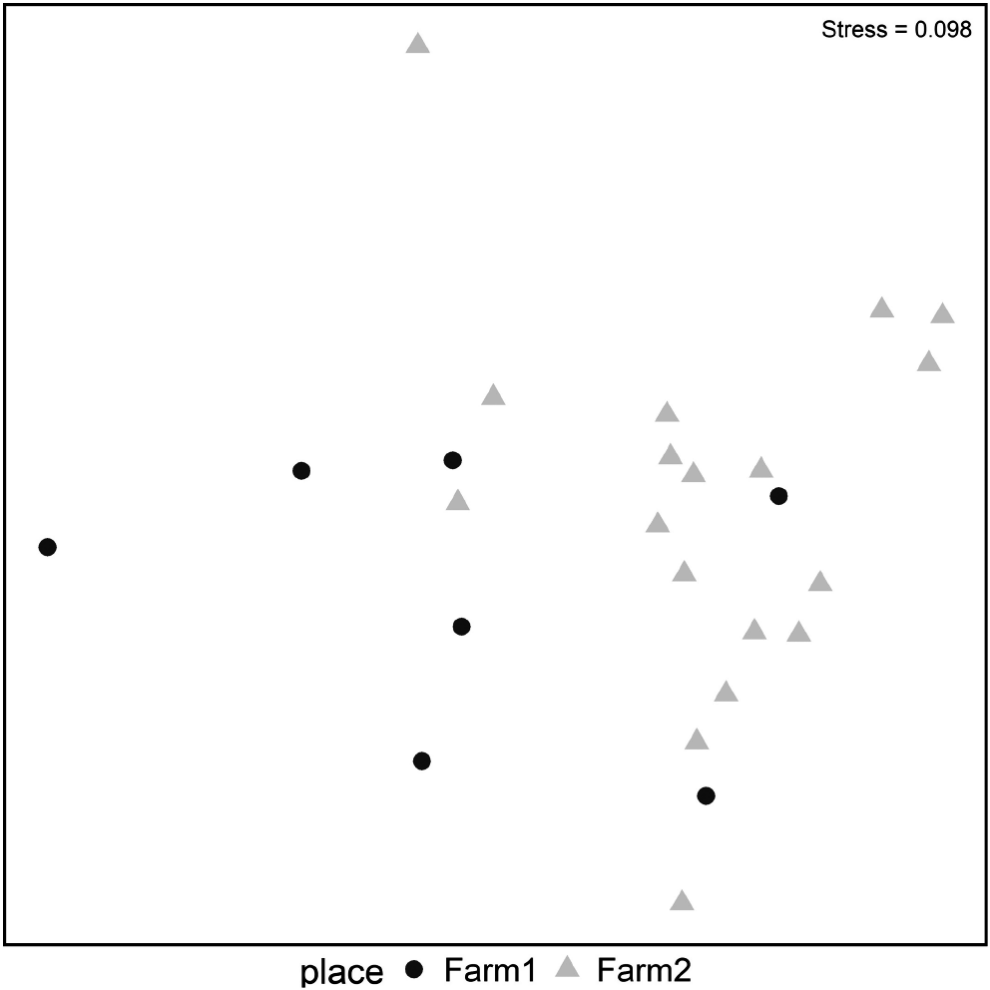
Visualization of helminth infracommunities using non-metric multidimensional scaling. Note: Each dot represents a sample from an individual horse. The distances among dots illustrate levels of qualitative and quantitative differences in infracommunities. Greater distance between two dots indicates more pronounced differences between those two infracommunities

The Mantel test showed a significant impact of the collection location (farm) on nematode infracommunities composition (*r* = 0.28, *p* = 0.03), indicating subtle differences between the farms. Conversely, the age and sex of the horses did not significantly affect the composition of the nematode infracommunities in this study, as evidenced by Mantel tests: *r* = -0.06, *p* = 1, and *r* = -0.03, *p* = 1, respectively. Further analysis using a model-based approach confirmed the significance of the collection site’s impact on differences in infracommunities (LRT = 57.66, p ≪ 0.001). Within this analytical approach, when evaluating the impact of individual nematode species on the differences, only *C. longibursatus* emerged as the species that significantly influenced these differences (*p* = 0.042). The visualization of infracommunity similarities revealed a weak clustering by collection site (Fig. 3), which corresponded with the results of the Mantel test.

### Assessment of Anthelmintic Efficacy against Strongylids

The faecal egg counts (FEC) before anthelmintic treatment for horses on Farm #1 ranged from 300 to 2,475 EPG, averaging 1032.1 EPG (SD = 734.5). On Farm #2, the FEC ranged from 450 to 4,250 EPG, averaging 1,379.2 EPG (SD = 991.7). A comparison of FECs between these two farms using the bootstrap t-test revealed no statistically significant difference (*p* = 0.816). The correlation analysis indicated a significant and moderate positive relationship between EPG value and the number of strongylid individuals per sample (Spearman’s rank correlation; *r* = 0.55, *p* = 0.004).

Faecal egg count reduction tests (FECRT) conducted at both farms indicated the substantial reduction of efficacy of FBZ against strongylids. Specifically, at Farm #1, the FECRT was 36.4% (from 0 to 83.3%); in Farm #2, the FECRT was 22.7% (from 0 to 94.6%), which was considered as the presence of strong BZ resistance on both farms. Reduction of IVM efficacy was not observed at either farm; on Day 14, after IVM treatment, strongylid eggs were not observed in faecal samples from any of the horses, indicating the FECRT of 100%.

## Discussion

This research is the first analysis explicitly focused on the strongylid parasite communities in domestic horses in Slovakia. Previous studies carried out by coprological methods assessed only the level of horse infection or determined the distribution of anthelmintic resistance in strongylids [47–50], identified only some cyathostomin species [46] or examined the strongylid community simultaneously in the Czech and Slovak Republics [40]. In our study, 24 strongylid species were found in horses from two farms in Eastern Slovakia. Approximately the same number of species (19–25 species) was found to parasitize domestic horses in neighbouring areas– in Poland and western Ukraine [22, 23, 28, 61, 62]. Previously, the horse strongylid communities in the same region had higher species richness– 35 strongylid species were documented in domestic horses in former Czechoslovakia [40], 30 species were found in Poland [20], 34 species were observed in domestic horses in Ukraine in 1960–1970th [8]. More recent studies conducted in Poland and western Ukraine [23, 62] showed a significant decrease in the species richness of the strongylid communities to 19–25 species or less, which we assumed to be associated with the massive use of highly effective anthelmintic drugs during last decades [29, 62]. As a result, most species of large strongylids previously found in domestic horses (*Strongylus* spp., *Triodontophorus* spp., *Oesophagodontus robustus*, *Craterostomum acuticaudatum*, *Bidentostomum ivashkini*) were eliminated from the strongylid community. Our results showed the same trends on horse farms in Slovakia. In the present study, only 5.6% of horses were infected with *Strongylus* spp.; two species of the genus *Triodontophorus* were found in 14.3–16.7% of horses; while in 1960th more than 71.4% of horses in Czechoslovakia were infected with *Strongylus* spp. and 33.8% with *Triodontophorus* spp. [40]. Thus, in this study, we could observe the same tendency of decreasing the proportion of large strongylids in the horse parasite community and the dominance of cyathostomins, as was indicated in recent decades worldwide [5, 6, 25–27; 30]. Also, we could observe an increase in the dominance of one of the most prevalent cyathostomin species, *C. catinatum* (the Berger-Parker dominance index of 0.41). We assume that this indicates gradual changes in the strongylid community under the influence of anthelmintic drugs.

The distribution of the strongylid species found in our study into ten prevalence classes revealed a multimodal strongylid community mode with dominant, subdominant, background, and rare species, which is typical for strongylids in the wild and domestic equids rarely treated with anthelmintics drugs [21, 24, 38, 64–66]. However, we could observe a tendency for the gradual destruction of the multimodal structure of the community (see Fig. 2) when the number of background species (with prevalence > 20–50%) was markedly reduced. As this tendency had been observed in strongylid communities of frequently dewormed horses [5, 21, 24, 66], we assumed that due to the massive use of highly effective anthelmintics in Slovak horse farms, the strongylid communities would gradually decrease, and its structure gradually becomes bimodal (so-called “core-satellite mode”). This phenomenon also could be connected with the spreading of anthelmintic resistance in cyathostomins registered in Slovakia before [48–50].

Additionally, we compared strongylid communities on two farms to analyze the influence of factors, such as age, sex, and collection location on the strongylid community. A significant moderate positive relationship between EPG value and the number of strongylid individuals per sample was recorded on both farms. Early studies performed in different countries observed no direct correlation between egg count (EPG) and the number of adult strongylids in a horse’s large intestine [4, 18, 67] due to variations in egg productivity of different strongylid species simultaneously parasitize a horse [68]. Other relationships might exist between these parameters [4]; however, more data are necessary to analyze these relations.

The level of horse infection (EPG value) and the average number of strongylid individuals found per sample differed on both farms (on Farm #1, horses were less infected than on Farm #2); however, these differences were not statistically significant. Moreover, *C. catinatum* was identified as the dominant species in both farms. Our analysis of the influence of age and sex on the strongylid communities showed that these two factors were not as essential as the effect of the collection location (farm), which was most probably connected with differences in horse management conditions as it was observed in other studies [5, 22, 24, 38, 62]. On the infracommunity level, the strongylid communities in horses from Farm #1 with no pasture access (only short-term access to individual paddocks) and more regular anthelmintic treatment significantly differed from infracommunities in horses from Farm #2 with less regular anthelmintic treatment and access to permanent pastures (see Fig. 3). One cyathostomin species, *C. longibursatus*, emerged as the species that significantly influenced these differences. This species was found to be the most widespread and abundant species of equine strongylids worldwide [6]. Even though almost nothing is known about the epidemiology and bionomics of separate cyathostomin species parasitize horses [13], we can assume that transmission of *C. longibursatus* is more dependent on horse access to pasture grazing than the transmission of other cyathostomin species. However, more data on the epidemiology and bionomics of separate cyathostomin species are necessary to fit the models that will test this assumption precisely.

Thus, our results present the first report on the strongylid community structure in domestic horses in Slovakia. Even though the study was performed on a small number of horses, it can be used as the basis for planning further studies of equine parasites in the region. New information on resistant strongylid species obtained from this study can potentially be used to extend our knowledge of strongylid epidemiology and will be useful for developing effective methods for horse parasite control in the country.

### Electronic Supplementary Material

Below is the link to the electronic supplementary material.

Supplementary Material 1

## Data Availability

The original datasets are available upon request to the corresponding author.
