# Supplementary material for: Strongylids of Domestic Horses in Eastern Slovakia: Species Diversity and Evaluation of Particular Factors Affecting Strongylid Communities
Source: Acta Parasitol. 2024 May 22;69(2):1284–94. doi: 10.1007/s11686-024-00854-7 (PMC11182797; doi:10.1007/s11686-024-00854-7)
Supplement: Supplementary file 1 — Supplementary Material 1 [file 11686_2024_854_MOESM1_ESM.pdf]

Supplement

The supplement provides an R code for the statistical tests employed in the study for the article titled *Strongylids of domestic horses in Eastern Slovakia: species diversity and evaluation of particular factors affecting strongylid communities.*

Load libraries

```
library(tidyverse)
library(magrittr)
library(MKinfer)
library(ecolTest)
library(vegan)
library(mvabund)

seed = 42
```

Data

the dataframe with the entire dataset

```
str(data)
```

```
tibble [25 x 31] (S3: tbl_df/tbl/data.frame)
 $ id              : chr [1:25] "2K" "3K" "4K" "5K" ...
 $ place           : Factor w/ 2 levels "Farm1","Farm2": 1 1 1 1 1 1 1 2 2 2 ...
 $ EPG             : num [1:25] 625 1475 2475 650 1000 ...
 $ age             : num [1:25] 4 6 3 7 7 4 6 2 1.5 8 ...
 $ age_cat         : chr [1:25] "1 - 4" "5 - 14" "1 - 4" "5 - 14" ...
 $ totalIntesity   : num [1:25] 134 3126 3569 396 398 ...
 $ sex             : chr [1:25] "M" "F" "M" "F" ...
 $ C.nasssatus     : num [1:25] 4 1461 608 187 67 ...
 $ C.catinatum     : num [1:25] 71 112 2144 185 169 ...
 $ C.insigne       : num [1:25] 0 116 86 2 8 3 0 0 0 0 ...
 $ C.leptostomum   : num [1:25] 0 717 66 4 30 0 0 53 333 2 ...
 $ C.calicatus     : num [1:25] 15 67 50 1 37 5 10 25 18 4 ...
 $ C.aswhorthi     : num [1:25] 0 25 48 0 0 8 0 32 164 4 ...
 $ C.pateratum     : num [1:25] 18 316 71 4 64 10 0 85 13 32 ...
 $ C.longibursatus : num [1:25] 15 29 243 11 7 35 2 83 13 243 ...
 $ C.goldi         : num [1:25] 3 40 27 2 6 7 0 2 0 0 ...
 $ C.minutus       : num [1:25] 0 60 36 0 4 0 0 14 98 327 ...
 $ C.coronatus     : num [1:25] 7 87 80 0 5 0 2 20 10 3 ...
```

```
 $ C.elongatus      : num [1:25] 0 0 0 0 0 0 0 0 0 0 ...
 $ C.radiatus       : num [1:25] 0 0 0 0 0 0 0 3 0 0 ...
 $ C.labiatus       : num [1:25] 0 73 101 0 0 0 0 9 29 9 ...
 $ C.labratus       : num [1:25] 0 18 4 0 0 0 0 0 9 0 ...
 $ P.poculatus      : num [1:25] 0 0 0 0 0 0 0 4 4 0 ...
 $ P.imparidentatum : num [1:25] 0 3 0 0 0 0 0 0 0 0 ...
 $ P.euproctus      : num [1:25] 0 1 0 0 0 0 0 0 0 0 ...
 $ P.mettami        : num [1:25] 0 1 2 0 1 0 2 1 0 0 ...
 $ C.bidentatus     : num [1:25] 1 0 0 0 0 0 0 0 0 0 ...
 $ C.brevicapsulatus: num [1:25] 0 0 0 0 0 0 0 0 0 0 ...
 $ S.vulgaris       : num [1:25] 0 0 0 0 0 0 0 0 0 0 ...
 $ T.serratus       : num [1:25] 0 0 1 0 0 0 0 0 1 0 ...
 $ T.brevicauda     : num [1:25] 0 0 2 0 0 0 0 0 0 0 ...
```

```
data %>% select(EPG, place) %>%
  datasummary(EPG * place ~ min+ max +mean +SD, data = ., dinm=FALSE)
```

preparing abundance matrix

```
abundanceMatrix  <- data %>% select(id, C.nasssatus : last_col()) %>%
  column_to_rownames(var='id')
class(abundanceMatrix)
```

```
[1] "data.frame"
```

preparing the list with data on separate farms

```
dataFarms <- data %>%
   select(id, place, C.nasssatus : last_col()) %>%
   column_to_rownames(var = 'id') %>%
   split(.$place) %>%
   map(~select(., -place)) %>%
   map(~select_if(., ~sum(.) > 0))
class(dataFarms)
```

```
[1] "list"
```

Univariate tests

performing univariate tests for comparing two farms

```
# species richness
richnessComparison <- wilcox.test(
    rowSums(dataFarms$Farm1 > 0),
    rowSums(dataFarms$Farm2 > 0),
    exact = F,
    paired=F) %>%
  print()
```

```
    Wilcoxon rank sum test with continuity correction

data:  rowSums(dataFarms$Farm1 > 0) and rowSums(dataFarms$Farm2 > 0)
W = 44.5, p-value = 0.2722
alternative hypothesis: true location shift is not equal to 0
```

```
# intensity
intensityComparison <- MKinfer::boot.t.test(
   totalIntesity ~ place,
   data = data,
   R = 50000,
   set.seed = 42) %>%
  print()
```

```
    Bootstrap Welch Two Sample t-test

data:  totalIntesity by place
bootstrap p-value = 0.02748
bootstrap difference of means (SE) = -2097.04 (757.5127)
95 percent bootstrap percentile confidence interval:
 -3569.2075  -577.9442

Results without bootstrap:
t = -2.5987, df = 17.457, p-value = 0.01845
alternative hypothesis: true difference in means is not equal to 0
95 percent confidence interval:
 -3792.7527  -397.5647
sample estimates:
mean in group Farm1 mean in group Farm2
           1138.286            3233.444
```

```
 # Shannon index
diversityComparison <- ecolTest::Hutcheson_t_test(
   colSums(dataFarms$Farm1),
   colSums(dataFarms$Farm2)) %>%
  print()
```

```
    Hutcheson t-test for two communities

data:  colSums(dataFarms$Farm1) ,  colSums(dataFarms$Farm2)
Hutcheson t-statistic = -0.19989, df = 10092, p-value = 0.8416
alternative hypothesis: true difference in H' is not equal to 0
sample estimates:
       x        y
1.804605 1.807201
```

```
 #EPG
epgComparison <-  MKinfer::boot.t.test(
   EPG ~ place,
```

```
    data = data,
    paired = FALSE,
    R = 50000,
    set.seed = 42) %>%
  print()
```

```
    Bootstrap Welch Two Sample t-test

data:  EPG by place
bootstrap p-value = 0.3856
bootstrap difference of means (SE) = -347.0766 (335.1957)
95 percent bootstrap percentile confidence interval:
 -1002.3909   334.3304

Results without bootstrap:
t = -0.95618, df = 14.883, p-value = 0.3543
alternative hypothesis: true difference in means is not equal to 0
95 percent confidence interval:
 -1121.1161   427.0685
sample estimates:
mean in group Farm1 mean in group Farm2
           1032.143            1379.167
```

adjust p-values for multiple comparisons

```
# Apply holm adjustment method
p.adjust(
    c(
      richnessComparison$p.value,
      intensityComparison$p.value,
      diversityComparison$p.value,
      epgComparison$p.value
    ),
    method = 'holm'
  ) %>%
round(3)
```

```
[1] 0.816 0.074 0.842 0.816
```

Estimate between correlation EPG and number of nematode individuals per sample

```
data %>%
  {
    cor.test(.$EPG,  .$totalIntesity, method = 'spearman', exact = F) %>%
    print()
  }
```

```
    Spearman's rank correlation rho

data:  .$EPG and .$totalIntesity
S = 1157.9, p-value = 0.004009
alternative hypothesis: true rho is not equal to 0
sample estimates:
      rho
0.5546575
```

Exploring relationships between the composition of communities and certain factors: Mantel Tests

performing Mantel tests

```r
set.seed(seed)

mantelPlace <- vegan::mantel(
    vegdist(abundanceMatrix, distance = "bray"),
    cluster::daisy(as.data.frame(data$place, stringsAsFactors = T),
                   metric = "gower"),
    method = 'spearman',
    permutations = 999
  ) %>%
  print()
```

```
Mantel statistic based on Spearman's rank correlation rho

Call:
vegan::mantel(xdis = vegdist(abundanceMatrix, distance = "bray"),      ydis = cluster::daisy(as.data.fra

Mantel statistic r: 0.285
      Significance: 0.01

Upper quantiles of permutations (null model):
  90%   95% 97.5%   99%
0.134 0.179 0.218 0.276
Permutation: free
Number of permutations: 999
```

```r
mantelAge <- vegan::mantel(
    vegdist(abundanceMatrix, distance = "bray"),
    vegdist(data$age),
    method = 'spearman',
    permutations = 999
  ) %>%
  print()
```

```
Mantel statistic based on Spearman's rank correlation rho

Call:
vegan::mantel(xdis = vegdist(abundanceMatrix, distance = "bray"),      ydis = vegdist(data$age), method

Mantel statistic r: -0.06075
      Significance: 0.717

Upper quantiles of permutations (null model):
  90%   95% 97.5%   99%
0.125 0.166 0.210 0.276
Permutation: free
Number of permutations: 999
```

```r
mantelSex <- vegan::mantel(
    vegdist(abundanceMatrix, distance = "bray"),
    cluster::daisy(as.data.frame(data$sex, stringsAsFactors = T), metric = "gower"),
    method = 'spearman',
    permutations = 999
  ) %>%
  print()
```

```
Mantel statistic based on Spearman's rank correlation rho

Call:
vegan::mantel(xdis = vegdist(abundanceMatrix, distance = "bray"),      ydis = cluster::daisy(as.data.fra

Mantel statistic r: -0.03509
      Significance: 0.828

Upper quantiles of permutations (null model):
   90%    95%  97.5%    99%
0.0517 0.0813 0.1114 0.1469
Permutation: free
Number of permutations: 999
```

adjust p-values for multiple comparisons

```r
# Apply holm adjustment method
p.adjust(
  c(
    mantelPlace$signif,
    mantelAge$signif,
    diversityComparison$signif,
    mantelSex$signif
  ),
  method = 'holm'
) %>%
round(3)
```

```
[1] 0.03 1.00 1.00
```

Model-based analysis of the composition of communities: comparison between farms

```
model <- mvabund::manyglm(mvabund(abundanceMatrix) ~ data$place, family = "negative_binomial")
plot(model)
```

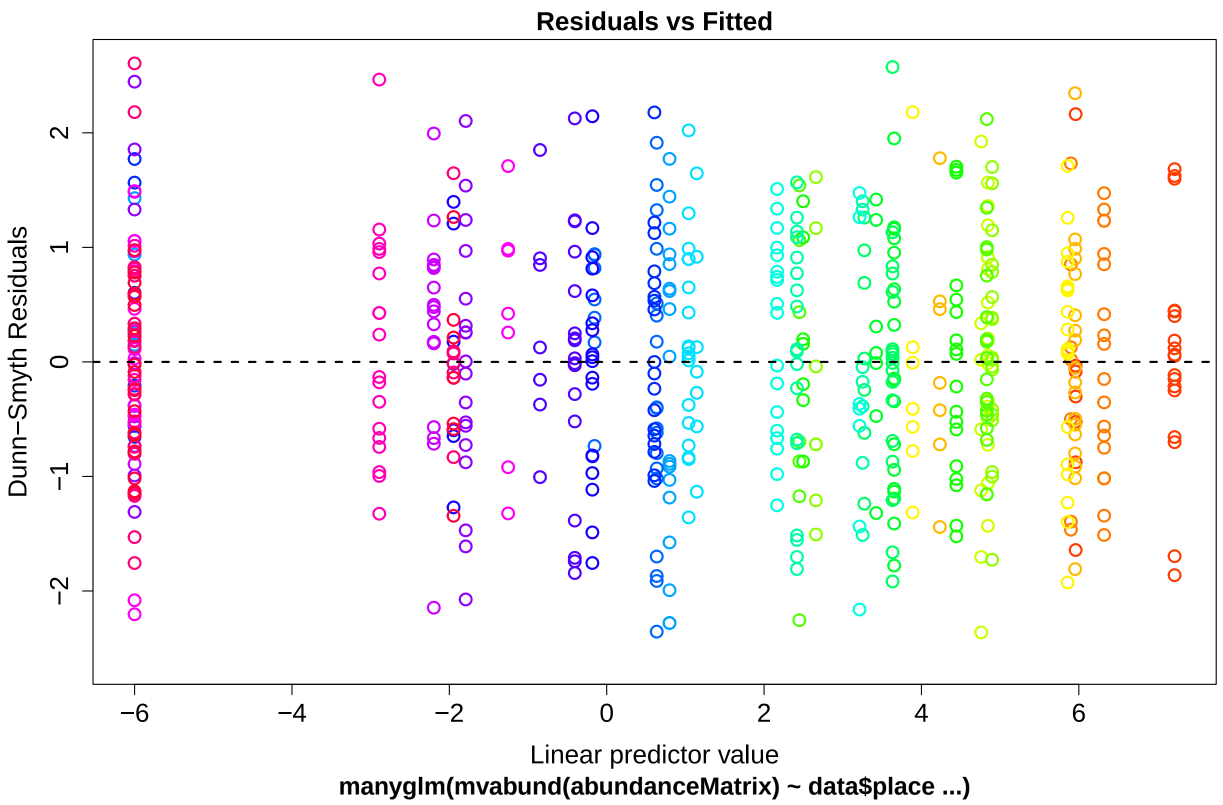


```
set.seed(seed)
mvabundAnova <- anova(
    model,
    test = 'LR',
    p.uni = "adjusted",
    nBoot = 10000,
    resamp = 'montecarlo'
  ) %T>%
  print()
```

```
Time elapsed: 0 hr 2 min 20 sec
Analysis of Deviance Table

Model: mvabund(abundanceMatrix) ~ data$place

Multivariate test:
            Res.Df Df.diff   Dev Pr(>Dev)
```

```
(Intercept)     24
data$place      23       1 57.66   <2e-16 ***
---
Signif. codes:  0 '***' 0.001 '**' 0.01 '*' 0.05 '.' 0.1 ' ' 1

Univariate Tests:
            C.nasssatus          C.catinatum          C.insigne
                    Dev Pr(>Dev)         Dev Pr(>Dev)       Dev Pr(>Dev)
(Intercept)
data$place        0.459    0.989       3.459    0.502     0.045    0.999
            C.leptostomum          C.calicatus          C.aswhorthi
                      Dev Pr(>Dev)         Dev Pr(>Dev)         Dev Pr(>Dev)
(Intercept)
data$place          0.011    0.999       0.385    0.989       4.945    0.347
            C.pateratum          C.longibursatus          C.goldi
                    Dev Pr(>Dev)             Dev Pr(>Dev)     Dev Pr(>Dev)
(Intercept)
data$place        4.483    0.374           8.732    0.043   4.917    0.347
            C.minutus          C.coronatus          C.elongatus
                  Dev Pr(>Dev)         Dev Pr(>Dev)         Dev Pr(>Dev)
(Intercept)
data$place      5.493    0.230       1.251    0.981       0.657    0.989
            C.radiatus          C.labiatus          C.labratus
                   Dev Pr(>Dev)        Dev Pr(>Dev)        Dev Pr(>Dev)
(Intercept)
data$place       0.683    0.989      1.186    0.981      0.008    0.999
            P.poculatus          P.imparidentatum          P.euproctus
                    Dev Pr(>Dev)              Dev Pr(>Dev)         Dev Pr(>Dev)
(Intercept)
data$place        7.211    0.102            0.024    0.999       2.546    0.801
            P.mettami          C.bidentatus          C.brevicapsulatus
                  Dev Pr(>Dev)          Dev Pr(>Dev)               Dev Pr(>Dev)
(Intercept)
data$place      0.734    0.989        2.546    0.726             3.691    0.502
            S.vulgaris          T.serratus          T.brevicauda
                   Dev Pr(>Dev)        Dev Pr(>Dev)          Dev Pr(>Dev)
(Intercept)
data$place       0.683    0.989      0.779    0.989        2.728    0.644
Arguments:
 Test statistics calculated assuming uncorrelated response (for faster computation)
P-value calculated using 10000 iterations via parametric resampling.
```

```r
mvabundAnova$uni.p %>%
  t() %>%
  as.data.frame() %>%
  filter(`data$place` < 0.05)
```

```
                (Intercept) data$place
C.longibursatus          NA  0.0429957
```

Non-metric Multidimensional Scaling

```
mds <- vegan::metaMDS(
    abundanceMatrix,
    distance = "bray",
    autotransform = F,
    trace = 0,
    set.seed = seed
  )
class(mds)
```

```
[1] "metaMDS" "monoMDS"
```
